# Maintenance of exercise training benefits is associated with adequate milk and dairy products intake in elderly hypertensive subjects following detraining

**DOI:** 10.1590/S1679-45082017AO4048

**Published:** 2017

**Authors:** Wilson Max Almeida Monteiro de Moraes, Neucilane Silveira dos Santos, Larissa Pereira Aguiar, Luís Gustavo Oliveira de Sousa

**Affiliations:** 1 Universidade de Fortaleza, Fortaleza, CE, Brazil.; 2 Universidade Estadual do Ceará, Fortaleza, CE, Brazil.; 3 Faculdade Nordeste - FANOR, Fortaleza, CE, Brazil.; 4 Universidade de São Paulo, São Paulo, SP, Brazil.

**Keywords:** Hypertension, Exercise, Elderly nutrition, Dairy products, Hipertensão, Exercício, Nutrição do idoso, Laticínios

## Abstract

**Objective:**

To investigate whether maintenance of exercise training benefits is associated with adequate milk and dairy products intake in hypertensive elderly subjects after detraining.

**Methods:**

Twenty-eight elderly hypertensive patients with optimal clinical treatment underwent 16 weeks of multicomponent exercise training program followed by 6 weeks of detraining, and were classified according to milk and dairy products intake as low milk (<3 servings) and high milk (≥3 servings) groups.

**Results:**

After exercise training, there was a significant reduction (p<0.001) in body weight, systolic, diastolic and mean blood pressure, an increase in lower and upper limb strength (chair-stand test and elbow flexor test) as well as in aerobic capacity (stationary gait test) and functional capacity (sit down, stand up, and move around the house) in both groups. However, in the Low Milk Intake Group significant changes were observed: body weight (+0.5%), systolic, diastolic and mean blood pressure (+0.9%,+1.4% and +1.1%, respectively), lower extremity strength (-7.0%), aerobic capacity (-3.9%) and functional capacity (+5.4) after detraining. These parameters showed no significant differences between post-detraining and post-training period in High Milk Intake Group.

**Conclusion:**

Maintenance of exercise training benefits related to pressure levels, lower extremity strength and aerobic capacity, is associated with adequate milk and dairy products intake in hypertensive elderly subjects following 6 weeks of detraining.

## RESUMO

**Objetivo:**

Investigar se a preservação dos benefícios do treinamento físico está associada à ingestão adequada de leite/produtos lácteos em idosos hipertensos após destreino.

**Métodos:**

Vinte e oito idosos hipertensos em tratamento clínico otimizado submetidos a 16 semanas de treinamento físico multicomponente, seguidas por 6 semanas de destreino, foram classificados segundo a ingestão de leite e produtos lácteos como baixo consumo (<3 porções/dia) e alto consumo (≥3 porções/dia).

**Resultados:**

Após o treinamento físico, houve redução significativa (p<0,001) em peso corporal, pressão arterial sistólica, diastólica e média, aumento na força de membros inferiores e superiores (testes sentar-levantar e flexão de cotovelos, respectivamente), capacidade aeróbica (teste da marcha estacionária) e capacidade funcional (sentar, levantar-se e mover-se pela casa) em ambos os grupos. Entretanto, no Grupo Baixo Consumo de Lácteos, observaram-se alterações significativas em peso corporal (+0,5%), pressão arterial sistólica, diastólica e média (+0,9%, +1,4% e +1,1%, respectivamente), força de membros inferiores (-7,0%), capacidade aeróbica (-3,9%) e capacidade funcional (+5,4) após o destreino. Estes parâmetros não apresentaram diferenças significativas entre o destreino e o período pós-treinamento no Grupo Alto Consumo de Lácteos.

**Conclusão:**

A preservação dos benefícios do treinamento físico em relação a pressão arterial, força de membros inferiores e capacidade aeróbia esteve associada com ingestão adequada de leite e produtos lácteos em idosos hipertensos após 6 semanas de destreino.

## INTRODUCTION

Cardiovascular diseases (CVD) are the number one cause of death worldwide and systemic arterial hypertension (SAH) is a clinical condition associated with high morbidity and cardiovascular mortality.^(^ ^1^ ^)^ Systemic arterial hypertension is one of the chronic diseases with higher prevalence in the elderly population and, in Brazil, more than half of them are considered hypertensive.^(^ ^2^ ^)^

Exercise training (ET) is a well-established non-pharmacological approach to prevent and treat SAH, reducing blood pressure (BP) and associated risk factors, such as obesity, insulin resistance and dyslipidemia.^(^ ^3^ ^)^ Moreover, multicomponent training (endurance, strength, coordination, balance and flexibility exercises) has shown be able to increase strength levels and attenuate a decline in physical function of older adults.^(^ ^4^ ^-^ ^6^ ^)^ However, the beneficial effects of ET may cease or regress when interruption of training periods occur; it is particularly important in older adults because they are more prone to episodes leading to ET interruption, such as severe trauma (*e.g*., brain injury or complicated fracture), have less adherence to training programs – in such situations, they present lack of training effects.^(^ ^4^ ^)^ Therefore, strategies to mitigate detraining effects may be interesting to attenuate or prevent the return to pre-training physiological conditions.

Some nutritional strategies, as dairy foods and high milk consumption showed an inverse association with incidence of CVD.^(^ ^7^ ^)^ One of the possible explanations for the beneficial effects of milk consumption on CVD health is its potential to lower BP, especially in individuals with elevated BP.^(^ ^8^ ^)^ There is also evidence that milk protein may indirectly improve metabolic health by enhancing lean body mass and skeletal muscle function, which may decrease progressive muscle mass loss, strength and function observed with aging.^(^ ^9^ ^)^

## OBJECTIVE

To investigate whether maintenance of exercise training effects after 6 weeks of detraining in elderly hypertensive patients is associated with adequate milk and dairy product intake (≥3 servings/ day).

## METHODS

### Subjects

Forty-four elderly subjects (>60 years) diagnosed with SAH and seen at a Primary Care Unit in city of Fortaleza, Ceará, Brazil, were recruited to participate in a quasi-experimental study. The objective was to assess the effects of a multicomponent ET program in functional capacity, physical fitness and drop in BP levels.^(^ ^6^ ^)^ The data were collected between August 2008 and February 2009, in the *Centro Comunitário Luiza Távora* - Secretariat of Labor and Social Development. All participants received the necessary information about the study and signed a consent form in accordance to National Health Council and Declaration of Helsinki. The research protocol was approved by the Research Ethics Committee of *Universidade de Fortaleza* (protocol 120/2007, CAAE: 1146.0.000.037-07 and Trial Registration RBR-2xgjh3). The exclusion criteria were: not completing 80% of all sessions and detraining evaluations; participation in another ET program; relevant calorie-intake restriction, inability to understand the instructions due to cognitive problems; uncontrolled hypertension (>160/100mmHg); chest pain; dizziness or discomfort; bone, muscle or joint problems, or any other previous condition that would preclude participation in the exercise program.

### Exercise training and detraining

**Figure 1:**
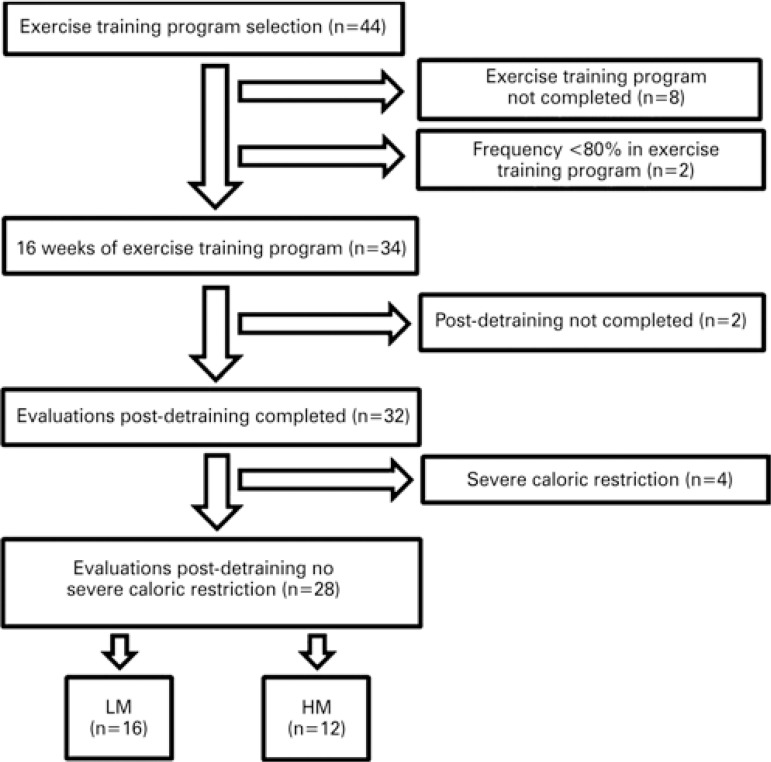
Schematic representation of volunteer participation

LM: Low Milk Intake Group; HM: High Milk Intake Group.

The ET program consisted of multi-component sessions twice a week, over a period of 16 weeks; the sessions lasted approximately 60 minutes as previously described.^(^ ^6^ ^)^ After 16 weeks, the ET program was discontinued for 6 weeks. Of 44 selected subjects, 8 left the ET program and 2 did not achieve 80% attendance. Hence, 34 patients completed the program; of these, two individuals did not complete detraining evaluations (Figure 1). In order to minimize the possible interfering effect of marked calorie intake restriction, the individuals presenting energy consumption below two standard deviations of the estimated energy expenditure (n=4) were excluded. Thus, 28 individuals were stratified in two groups according to the reported intake of dairy products: the Low Milk Intake Group (LM), comprising those with inadequate milk and dairy product intake (<3 portions/day) and High Milk Intake Group (HM), for those with milk and dairy products intake in accordance to current recommendations (≥3 portions/day)^(^ ^10^ ^)^ (Figure 1).

Blood pressure levels, body weight and motor tests were measured on three occasions: pre-training, after 16 weeks of training, and after six weeks of detraining. Dietary data were gathered after six weeks of detraining.

### Measurements

For height measurement, a stadiometer (Altura Exata Brazil) with 1mm precision was used; and, for body weight, a scale Plenna^®^, with precision of 100g. Body mass index (BMI) was calculated by the equation body mass (kg)/height^2^ (m^2^). Blood glucose, total cholesterol (TC), Low Density Lipoprotein (LDL)-cholesterol and triglycerides levels were obtained from individual registration forms.

The BP measurement was determined with a Missouri^®^ column sphygmomanometer and a Rappaport^®^ stethoscope, with the participant in the sitting position, uncrossed legs, feet flat on the floor. The mean of three measurements taken from the left arm was considered for analysis.^(^ ^11^ ^)^ The test-retest analysis showed an intra-rater correlation coefficient exceeding 0.85 for systolic BP (SBP) and diastolic BP (DBP).^(^ ^8^ ^)^ The mean BP (MBP) was calculated utilizing the formula: MBP = DBP + (SBP − DBP)/3.

For aerobic capacity measuring, the stationary gait test was used (SGT),^(^ ^12^ ^)^ in which the participant initiated knee flexion, simulating gait while standing in place, and the result was the number of steps taken during a 2 minute period.

Lower and upper limb muscle strength was determined by the chair-stand test (CST) and elbow flexor test (EFT), respectively.^(^ ^12^ ^)^ For CST, the individual started the test sitting a chair with a seat height of 43cm, arms crossed on the chest. For EFT, the participant sat on a chair, performed elbow flexion and extension cycles holding a dumbbell (2 and 4kg for women and men, respectively). The results were the total number repetitions performed in 30 seconds.

To assess functional ability, the sit down, stand up, and move around the house test (SSMT)^(^ ^13^ ^)^ were used. The SSMT consisted of walking around two cones twice alternating turns to the right and to the left. All participants knew the motor tests before performing them.

### Dietary data

A food frequency questionnaire previously developed was used,^(^ ^14^ ^)^ with local applicability in order to evaluate the energy and nutrient intake. The amounts were recorded in household measures to assist in the conversion of food amount described for grams and portions. The food consumption data were processed with DietWin^®^ software.^(^ ^15^ ^)^ The calcium, sodium and macronutrients intake was compared to current recommendations.^(^ ^10^ ^,^ ^16^ ^,^ ^17^ ^)^

The estimated energy requirement (EER) was based on equation EER = 662 - (9.53 x age) + [PA x (15.91 x weight + 539.6 x height)], in which PA corresponds to physical activity level.^(^ ^17^ ^)^

### Statistical analysis

Repeated-measures Multivariate Analysis of Variance (MANOVA) was used to examine differences within and between groups over time. Post-hoc comparisons were made with least significant difference test for multiple comparisons, when p≤0.05.

In addition, to check if adequate intake of dairy products during detraining could be associated with maintenance of gains observed with ET program, the delta percentage (%) was calculated using the standard formula: delta % = [(post-detraining score – post-exercise score)/pretest score]/100.

## RESULTS

The mean age in LM and HM groups was 70.2±4.9 and 70.3±5.0 years, respectively. The basal values for both groups were, respectively, for BMI 26.2±2.7kg/m^2^ and 25.5±2.3kg/m^2^; blood glucose of 109.5±15.3mg/dL and 107.4±14.6mg/dL; LDL-cholesterol 108.2±13.7mg/dL and 111.7±10.3mg/dL; triglycerides of 164.3±26.4mg/dL and 158.2±24.7mg/dL. Female participants accounted for 71.4% (n=20). The clinical characteristics are presented in table 1.

**Table 1:** Baseline clinical characteristics of participants

Parameter	Low Milk Intake Group n (%)	High Milk Intake Group n (%)
Dyslipidemia	8 (28.6)	6 (21.4)
Overweight*	7 (25)	6 (21.4)
*Diabetes mellitus* type 2	4 (14.3)	4 (14.3)
Alcoholism*	1 (3.6)	2 (7.1)
Smokers	3 (10.7)	3 (10.7)
Pharmacological treatment		
ACEIs	11 (39.3)	13 (46.4)
Angiotensin II AT1 receptor blockers	2 (7.1)	1 (3.6)
Beta blockers	3 (10.7)	3 (10.7)
Diuretics	6 (21.4)	5 (17.9)
Calcium channel blockers	1 (3.6)	2 (3.6)
Statins	6 (35.7)	4 (35.7)
Aspirin	1 (3.6)	1 (3.6)
Oral hypoglycemic drugs	6 (21.4)	5 (17.9)

* BMI>27kg/m^2^.

ACEIs: angiotensin-converting enzyme inhibitors; AT1: angiotensin receptor.

In post-detraining period, the mean milk and dairy products intake by the LM group (1.7 portion/day) was significantly lower than that reported by HM group (3.3 portions/day). Likewise, the calcium intake reported by LM group (704mg/day) was lower than the HM group (1,396mg/day). Moreover, 100% of the individuals in HM group consumed calcium above the Recommended Dietary Allowance (RDA)^(^ ^16^ ^)^ indicating that main source of calcium intake is through the consumption of milk and dairy products. All subjects consumed RDA values for proteins (at least 0.8g of protein/kg),^(^ ^17^ ^)^ and protein intake was not significantly different between the groups (0.93g/kg in LM and 1.19g/kg in HM), suggesting that the results were not attributed to total protein intake between the groups.

The energy intake reported in LM and HM were 1594.2±126.4kcal and 1628.4±141.2kcal, respectively, and showed no significant difference between groups (p>0.05). The carbohydrate intake amounted to 241.0g (56.5% total energy) in LM and 238.1g in HM (54.7% total energy). Lipid intake 51.3g (27.1% total energy) in LM and 49.1g (24.1% total energy) in HM (p>0.05), and sodium intake reported by LM was 1,637mg in LM and 1,724mg in HM (p>0.05).

Table 2 shows the results for body mass, BMI, BP levels and motor tests at pre-training, post-training and post-detraining for LM (n=16) and HM (n=12). The pre-training values for each variable did not differ between groups. After 16-week ET, there was significant improvement (p<0.05) in lower and upper extremity strength evaluated by CST and EFT respectively. In both groups the aerobic and functional capacity were better in SGT and SSMT (p<0.05).

**Table 2:** Body mass, body mass index, blood pressure levels and motor tests during pre-training, post training and post detraining in Low Milk Intake and High Milk Intake groups

Parameters	Pre-training		Post-training		Post-detraining	
		
	LM	HM	LM	HM	LM	HM
Body mass, kg	70.1±7.6	68.6±7.7	69.7±7.4*	68.0±6.1*	70.1±7.5*^†^	68.1±6.2*
BMI, m/kg^2^	26.2±2.7	25.5±2.3	26.1±2.7*	25.3±2.2*	26.2±2.7*^†^	25.3±2.2*
SBP, mmHg	137.6±3.6	138.3±4.6	134.7±4.0*	135.2±4.5*	135.9±3.6*^†^	135.8±4.7*
DBP, mmHg	90.1±4.4	91.3±5.3	87.5±4.3*	88.3±4.9*	88.8±4.3*^†^	88.8±5.0*
MBP, mmHg	105.9±3.1	103.7±5.7	103.2±3.2*	103.8±2.8*	104.4±3.0*^†^	104.2±2.9*
CST, repetitions	10.3±2.6	9.6±2.1	11.2±2.0*	12.0±1.8*	10.4±2.0*^†^	11.5±1.2*
EFT, repetitions	11.5±2.4	10.8±1.6	13.5±2.1*	13.0±1.4*	13.2±1.9*	12.7±1.3*
SGT, steps	71.7±9.7	72.4±9.2	78.1±10.2*	79.5±8.8*	75.1±9.7*^†^	77.8±8.6*
SSMT, seconds	42.1±6.9	42.0±8.0	38.2±5.0*	38.0±6.3*	40.2±6.0*^†^	38.6±6.4*

Values expressed as mean±standard deviation. * significant difference *versus* pre-training; p<0.001; ^†^ significant difference *versus* post-training, p<0.001.

LM: Low Milk Intake Group; HM: High Milk Intake Group; BMI: body mass index; SBP: systolic blood pressure; DBP: diastolic blood pressure; MBP: mean blood pressure; CST: chair-stand test; EFT: elbow flexor test; SGT: stationary gait test; SSMT: sit down, stand up, and move around the house.

After 6 weeks of detraining, body mass, BMI, BP levels, lower extremity strength, aerobic and functional capacity were worse as compared to post-training period in the LM group, as evidenced by increase in body mass, BMI, SBP, DBP, MBP as well as a drop in CST, SGT and SSMT. However, in HM group, no differences were observed between post-training and post-detraining periods regarding body mass, BMI and BP levels, and in CST, SGT and SSMT. There was no significant relation between changes in motor tests and BP with baseline body mass or BMI. These data suggest that adequate milk intake can attenuate or reduce loss of benefits brought by ET, such as improved body mass, BP levels, lower extremity strength, aerobic and functional capacity. As to upper limb strength, no significant changes were observed between post-training and post-detraining in both groups, as verified for EFT (p>0.05).

**Figure 2:**
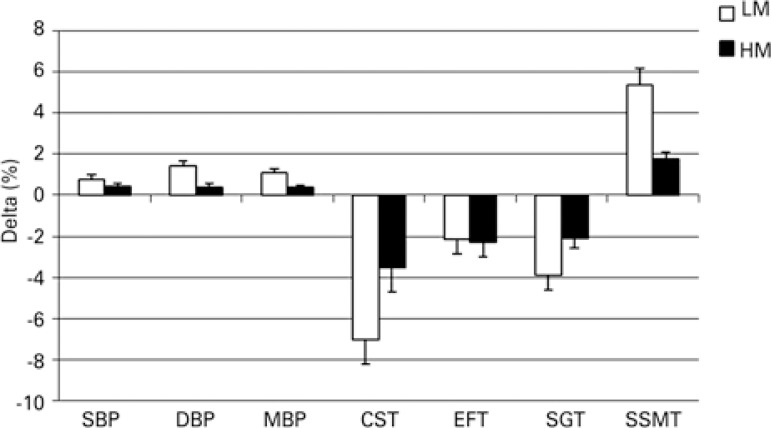
Relative mean changes in blood pressure levels and motor tests results between post-training period and 6-week detraining and in Low Milk Intake and High Milk Intake groups

Since the primary objective of the present study was to investigate the effects of milk and dairy products intake on detraining, the data related to nutritional intake were collected in the post-detraining period. As observed in figure 2, the relative changes that occurred between post-training period and 6 week-detraining were more pronounced in LM Group for all parameters, with exception of EFT results. These data corroborate the findings shown in table 2, and suggest that adequate milk and dairy products intake can help maintain gains achieved with ET.

Data are presented as mean±standard deviation.

LM: Low Milk Intake Group; HM: High Milk Intake Group; SBP: systolic blood pressure; DBP: diastolic blood pressure; MBP: mean blood pressure; EFT: elbow flexor test; SGT: stationary gait test; SSMT: sit down, stand up, and move around the house.

## DISCUSSION

The major finding of this study was that elderly hypertensive subjects were able to maintain gains achieved with 16 weeks of multicomponent training after 6 weeks of detraining, when they consumed milk and dairy products in accordance to the Food Guide for the Brazilian Population.^(^ ^10^ ^)^ These effects were observed in BP levels, lower limb strength and aerobic and functional capacity. The maintenance of exercise-induced hypotensive effects can help control hypertension and improve muscle strength and aerobic capacity, resulting in less difficulty in carrying out daily activities and better quality of life.

The appropriate intake of dairy products (≥3 servings/day), in special as part of the Dietary Approaches to Stop Hypertension (DASH) plan, has demonstrated a beneficial role in BP control.^(^ ^18^ ^)^ The main mechanisms involve the significant contribution for daily requirements of protein and calcium intake, as well as the presence of bioactive peptides.^(^ ^19^ ^)^ The calcium intake can influence multiple mechanisms involving calcitrophic hormones, vascular reactivity, intracellular calcium levels, as well as interaction with the renin-angiotensin system.^(^ ^19^ ^,^ ^20^ ^)^ The bioactive peptides act mainly in renin-angiotensin system, have antioxidant properties and interfere in blood lipids levels.^(^ ^19^ ^)^

The results of present study corroborate the fact that it is difficult to maintain adequate calcium levels when the milk and dairy products intake is lower than 3 servings/day.^(^ ^21^ ^)^ This is of particular interest since the mean calcium intake in elderly individuals is 527mg/day, and approximately 90% of elderly population in Brazil has calcium intake below RDA values.^(^ ^22^ ^)^

Moreover, the maintenance of gains related to motor tests in HM group after detraining period suggests some beneficial effect of adequate intake of milk and dairy products on musculoskeletal health. Although the amount of protein ingested is similar between groups, it is known that proteins with high biological value, especially milk-derived proteins, are able to improve single fiber contractile properties by optimization in protein synthesis, resulting in enhanced muscle strength and function.^(^ ^9^ ^)^

The improvement in mechanical muscle function induced by ET often leads to improved functional capacity in elderly individuals performing activities of daily living.^(^ ^9^ ^)^ The SSMT emphasizes deambulation, and as an indicator of functional capacity, suggested that HM maintained gains acquired with ET; this was probably influenced by maintenance of gains in lower extremity strength and aerobic capacity. This aspect is of particularly importance since hypertensive elderly patients are 4.2-fold more likely to develop functional limitations than those who are not hypertensive.^(^ ^23^ ^)^

Unlike what was observed in lower extremity strength, the upper limb strength remained significantly higher after 6 weeks of detraining as compared to pre-training in both groups. These results are corroborated by previous studies of elderly people with same detraining duration and weekly frequency.^(^ ^4^ ^)^ This suggests that exercise interventions may have a differential impact in distinct muscle groups, probably due to the characteristics of training sessions, with considerable utilization of upper extremities, as well as to the total duration of exercise program, which was longer in our study (16 weeks) in comparison to nine weeks in the investigation by Toraman.^(^ ^4^ ^)^

It is estimated that 20 to 25% of hypertensive individuals do not present lower BP levels after exercising. This heterogeneity of individual response to the antihypertensive effects of exercise is mostly attributed to genetic components and polymorphisms.^(^ ^24^ ^)^ However, the genetic components are not enough to explain this variance, and there is evidence suggesting that ingestion of nutrients and dietary components may contribute to this heterogeneous response to exercise.^(^ ^20^ ^)^ In the present study, the influence of milk products intake on BP levels could also be observed in detraining periods. Thus, dietary components should be further investigated regarding their possible interference in the antihypertensive responses to exercise.

The limitations of our study should be acknowledged. Since the data related to nutritional intake were collected in post-detraining period only, we cannot ensure that individuals maintained the same dietary habits during all ET training and detraining period. Future studies might consider data collection related to diet intake more frequently, with a more rigid control in variables interfering in BP levels.

## CONCLUSION

In summary, we provide evidence that maintenance of exercise training benefits is associated with adequate milk and dairy products intake (≥3 servings/day) in elderly hypertensive, reducing the increase in blood pressure levels and loss of lower limb strength and aerobic capacity, which results in better maintenance of functional capacity after six weeks of detraining.
